# High-Sensitivity NO_2_ Gas Sensor: Exploiting UV-Enhanced Recovery in a Hexadecafluorinated Iron Phthalocyanine-Reduced Graphene Oxide

**DOI:** 10.1021/acsomega.4c08662

**Published:** 2025-01-16

**Authors:** John A. Cruz Lozada, Ricardo A. Rosario, Soraya Y. Flores, Kim Kisslinger, Luis F. Fonseca, Dalice M. Piñero Cruz

**Affiliations:** † Faculty of Natural Sciences, University of Puerto Rico, Río Piedras Campus, San Juan 00931, Puerto Rico; ‡ Molecular Science Research Center, San Juan 00926-2614, Puerto Rico; # Center for Functional Nanomaterials, Brookhaven National Laboratory, Bldg 735 Upton New York 11973-5000, United States

## Abstract

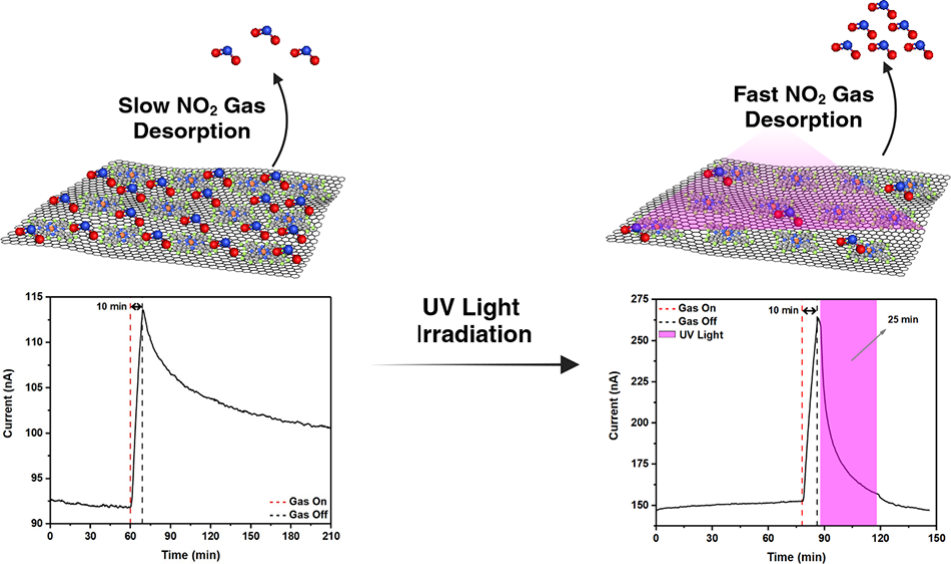

Monitoring ultralow nitrogen dioxide (NO_2_) concentrations is crucial for air quality management and public health. However, the existing NO_2_ gas sensors have several defects, like high cost and power consumption, and exhibit poor selectivity. This study addresses these challenges by presenting a novel hexadecafluorinated iron phthalocyanine-reduced graphene oxide (FePcF_16_-rGO) covalent hybrid sensor for NO_2_ detection. This innovative approach, which overcomes the limitations of fabrication cost, energy efficiency, and gas selectivity, is a significant step forward in gas sensor technology. The sensor demonstrates exceptional sensitivity toward ultralow NO_2_ concentrations (15.14% response for 100 ppb) with a rapid 60 s UV light-induced recovery. Additionally, the sensor exhibits high selectivity for NO_2_, achieving a limit of detection (LOD) of 8.59 ppb. This approach paves the way for developing cost-effective, energy-efficient, and miniature NO_2_ monitoring devices for improved environmental monitoring and enhanced safety in workplaces where NO_2_ exposure is a concern.

## Introduction

Toxic gases are often odorless and colorless; even very low concentrations threaten human health. For this reason, advanced gas sensors are required to trace air pollutants.^1^ Although effective, traditional environmental sensors, such as mass spectroscopy, gas chromatography, and Fourier transform infrared spectroscopy, have many drawbacks, such as high energy consumption, bulkiness, complexity in data analysis, and interference due to other gases and humidity.^2^ New sensor technologies include electrochemical, acoustic, nondispersive infrared, and organic-based chemiresistive sensors, among others.^3^ These new sensor technologies can overcome these deficiencies with enhanced precision and efficiency for air quality monitoring, workplace safety, and medical diagnosis applications.^4,5^ The innovations enable the detection of harmful gases, such as nitrogen dioxide (NO_2_), cost-effectively and with energy efficiency. NO_2_, mainly emitted by fossil fuel combustion, is a toxic pollutant to human health and the environment.^6,7^ It leads to severe respiratory disorders due to long-term exposure, even at low part per billion (ppb) levels, and contributes to ozone formation and acid rain.^8−11^ Effective NO_2_ sensors are required to mitigate the risks. Still, while sensitive, conventional metal oxide semiconductor sensors require high operating temperatures and suffer from power inefficiency, selectivity, and stability.^12−15^ Advances in gas sensing technology aim to develop high-performance sensors that can detect NO_2_ at room temperature, which is a significant move toward practical and portable devices.

The realization of this critical need has seen the growth of different gas sensing technologies, with each sensing technology trying to solve specific problems that occur with detecting a particular gas. For example, it has been observed that conducting polymer composites, metal–organic frameworks, and carbon-based nanomaterials have great sensitive and versatile performance with a wide range of toxic gas detection.^16−18^ Many such materials can work at room temperature, which makes these sensors energy-efficient and suitable for portable applications. However, material and sensor design strongly influence their selectivity to certain gases. For example, Duan et al. prepared a polyaniline (PANI) composite with halloysite nanotubes (HNTs) using a simple in situ polymerization method to enhance the performance of ammonia (NH_3_) gas sensing. The PANI/HNTs sensor exhibited enhanced sensitivity, 7.26% ppm^–1^ for 0.1–10 ppm NH_3_, a higher response, 91.99% to 10 ppm NH_3_, and faster response/recovery times, 158/169 s, which shows how one can optimize a gas sensor for a single toxic gas.^19^

The specific detection of NO_2_, a highly adverse and health-threatening pollutant, takes other material considerations into account. NO_2_ is chemically reactive and often coexists with other gases, making selectivity and stability critical parameters for sensors. For this reason, recent advances in nanostructured materials, such as transition metal carbides/nitrides, nanoparticles, and graphene-based hybrid materials, have addressed some challenges by offering enhanced selectivity and sensitivity at room temperature.^20,21^ For example, transition metal carbides/nitrides have excellent electrical conductivity and chemical stability with a high surface area, which could enable the effective detection of NO_2_ through their unique layered structure and abundant active sites.^22^ Similarly, graphene-based materials are also very suitable for exceptional sensing due to their high surface-to-volume ratio, robust conductivity, and versatile functionalization possibilities, rendering both materials suitable for NO_2_ sensing at room temperature.

Graphene nanosheet-based gas sensors, especially those based on graphene oxide (GO) and reduced graphene oxide (rGO), have emerged as new technologies featuring high specific surface area, chemical stability, and tunable optical and electrical properties.^23,24^ Since the pioneering work of Novoselov’s group in 2007, showing conductivity changes of graphene upon exposure to gas molecules.^25^ rGO has exhibited promising selectivity toward NO_2_ and other gases. Other works, such as incorporating rGO into electrospun nylon-6 fibers by Park et al. and the thermal annealing processes by Zhou et al., have reported improved flexibility, stability, and gas sensing performance.^26,27^ Hybrid structures, such as rGO/tungsten disulfide (WS_2_) heterojunctions, have also exhibited improved room-temperature responses and stability.^28^ Nevertheless, several limitations involving rGO-based sensors include solubility, recovery time, and selectivity.^29,30^ Functionalization or doping of rGO, especially with materials like metal phthalocyanines (MPs), has efficiently enhanced sensitivity and selectivity.^31^ MPcs represent versatile organic semiconductors, having a planar macrocycle structure with a tunable central metal ion that can be adjusted to show p-type or n-type semiconductor behavior in targeted gas detection.^32−34^ These structural and electronic properties, combined with MPcs’ ability to interact extensively with gas molecules, have been exploited to detect pollutants like NH_3_, hydrogen sulfide (H_2_S), chlorine (Cl_2_), and volatile organic compounds (VOCs).^35−38^ For example, Guo et al. reported enhanced sensitivity to NH_3_ by hybridizing CoPc with rGO, while Kumar et al. used MPcs with rGO for Cl_2_ detection, illustrating the potential of rGO-MPc hybrids for high-performance gas sensing.^39,40^

Recent research in our laboratory has explored the fabrication and application of MPc-based nanowire sensors for gas detection. Otero et al. focused on developing a palladium phthalocyanine nanowire sensor for detecting NO_2_ at subppm levels, demonstrating its effectiveness at room temperature with a notable response even at 0.5 ppm.^41^ Flores et al. investigated the use of fluorinated iron and cobalt phthalocyanine nanowire sensors for environmental gas monitoring, particularly for detecting NH_3_ in the ultralow ppb range, emphasizing their suitability for long-term monitoring in recovery zones due to their low power consumption and room-temperature operation.^35^ Inspired by these promising results, we present a novel approach to NO_2_ sensing by developing a nanohybrid material based on hexadecafluorinated iron phthalocyanine (FePcF_16_) and rGO. This innovative hybrid structure aims to leverage the synergistic properties of both components to achieve exceptional sensitivity, selectivity, and rapid recovery for NO_2_ detection, particularly at low concentrations relevant to environmental monitoring and workplace safety. Moreover, we anticipate that the incorporation of rGO will significantly improve the conductivity of the sensing material, allowing for the fabrication of high-current sensors that offer benefits such as enhanced signal strength and faster response times.

## Experimental Section

### Materials

All chemicals and reagents used in the study were sourced commercially and employed without further purification. Dehydrated N, N-dimethylformamide (DMF), acetone, ethanol, *n*-hexane, and graphite were obtained from Sigma-Aldrich and utilized without additional purification. Tetrafluorophthalonitrile (PnF_4_) and iron(II) acetate (Fe(OAc)_2_) were acquired from Fisher Scientific. GO, FePcF_16,_ and FePcF_16_-rGO hybrid were synthesized according to the literature.^31,35,39^

### Synthesis of FePcF_16_

A mixture of PnF_4_ (500 mg; 2.5 mmol) and Fe(OAc)_2_ (435 mg; 2.5 mmol) was added in a 25 mL Teflon liner. The Teflon liner was capped inside an argon drybox and placed inside a reactor, then removed and placed inside an oven and heated to 250 °C for 5 h. The reaction was left inside the oven until it reached room temperature; the obtained dark-violet solid was pulverized using a mortar and pestle. The resulting powder was placed in boiling hexane to remove any unreacted phthalonitrile, then filtered and washed with nanopure water to remove unreacted Fe(OAc)_2_. Afterward, the crude dry powder was extracted for 2 days with a Soxhlet apparatus using dry acetone that gave a blue liquid. The collected liquid was roto evaporated, thus affording a dark-violet solid that was oven-dried at 75 °C.

### Preparation of the FePcF_16_-rGO Hybrid

The FePcF_16_-rGO hybrid was prepared by hydrazine reduction of GO in the presence of the FePcF_16_. GO was prepared using a modified Hummer method.^31,39^ GO (100 mg) was sonicated in a Schlenk tube in DMF for 2 h to form a homogeneous dispersion. The FePcF_16_ (200 mg) was dissolved in DMF, added dropwise to GO dispersion, and sonicated for 2 h. Afterward, hydrazine and ammonia–water were added. The Schlenk tube containing the resulting mixture was then placed in an oil bath at 100 °C and stirred for 24 h under a nitrogen atmosphere. The solution was cooled and filtered, then washed with DMF, ethanol, and acetone in sequence until the filtrate was colorless. The resulting black powder was transferred into a vial and placed in a vacuum oven at 60 °C for 24 h.

### Characterization

UV/vis absorption spectra were recorded using a Shimadzu UV-1800 (Kyoto, Japan). FT-IR was recorded on a Nicolet iS50 (Rochester, NY, USA). The Raman spectra were obtained using a Raman spectrophotometer (HR800, HORIBA JobinYvon Company) exploited by a laser with a 457.9 nm wavelength. The materials’ scanning electron microscopy (SEM) images were obtained using a Phenom Pharos G2 Desktop FEG-SEM (Thermo Fisher Scientific Corporation). The transmission electron microscopy (TEM) work was done using a FEI F200X.

### Gas Sensor Fabrication and Sensing Measurements

A drop-casting technique was employed to fabricate the gas sensor. This involved preparing a 1.0 mg/mL dispersion of the FePcF_16_-rGO hybrid in ethanol. The mixture was sonicated for 2 h, and then 50 μL was dispersed on top of gold interdigitated electrodes (IDE). The solvent was then evaporated, followed by placing the sensor in a vacuum oven for 5 h at 80 °C to remove residual solvent. The same procedure was applied to the rGO and FePcF_16_ gas sensors.

The gas sensor testing procedure, adapted from a previously published protocol by our laboratory, involved placing the prepared sensor in an MMR Technologies LTMP gas testing chamber, electrically connecting it with tungsten tips, and testing it at room temperature (24 ± 1 °C) using a Keithley 6487 Picoammeter/Voltage Source to record current every 15 s, while exposing it to controlled nitrogen and NO_2_ mixture, regulated by MKS GE50A Mass Flow Controllers at a total flow rate of 500 sccm and monitored by an MKS 946 Vacuum System Controller.^35^

Equations 1 and 2 were used to characterize the gas sensor’s response at room temperature (RT) and its ability to recover to its initial state upon gas exposure and removal:^42^
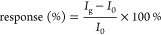
1
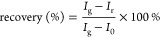
2where *I*_0_ is the initial current intensity before the exposure to the pollutant gas, *I*_g_ is the current upon exposure to the pollutant gas at a certain time “*t*”, and *I*_r_ is the recovery current at a specific time. The light source in the experiment was a UV light-emitting diode (LED) with a wavelength of 365 nm and a power output of 18.4 W.

## Results and Discussion

### Synthesis and Characterization of the FePcF_16_-rGO Hybrid

**Scheme 1:**
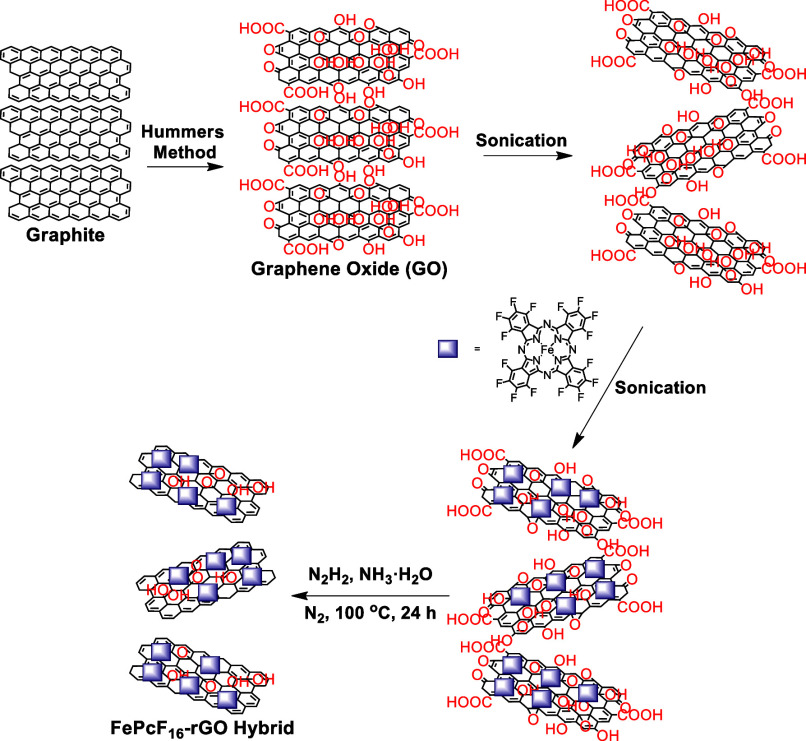
Schematic Illustrating the Synthesis of the FePcF_16_-rGO Hybrid The process involves oxidizing graphite to graphene oxide via a modified Hummers method, anchoring FePcF_16_ onto the GO surface, and reducing it with hydrazine to form the final hybrid material.

We report the successful synthesis of a novel nanohybrid material, integrating FePcF_16_ and rGO, representing a significant advancement in the field. To synthesize FePcF_16_, we employed a modified procedure reported by our research group where the cyclotetramerization of PnF_4_ is achieved via a solid-state reaction.^35^ Following an established procedure adapted to incorporate the unique properties of FePcF_16_, the FePcF_16_-rGO hybrid was prepared, as illustrated in Scheme 1.^43^

**Figure 1:**
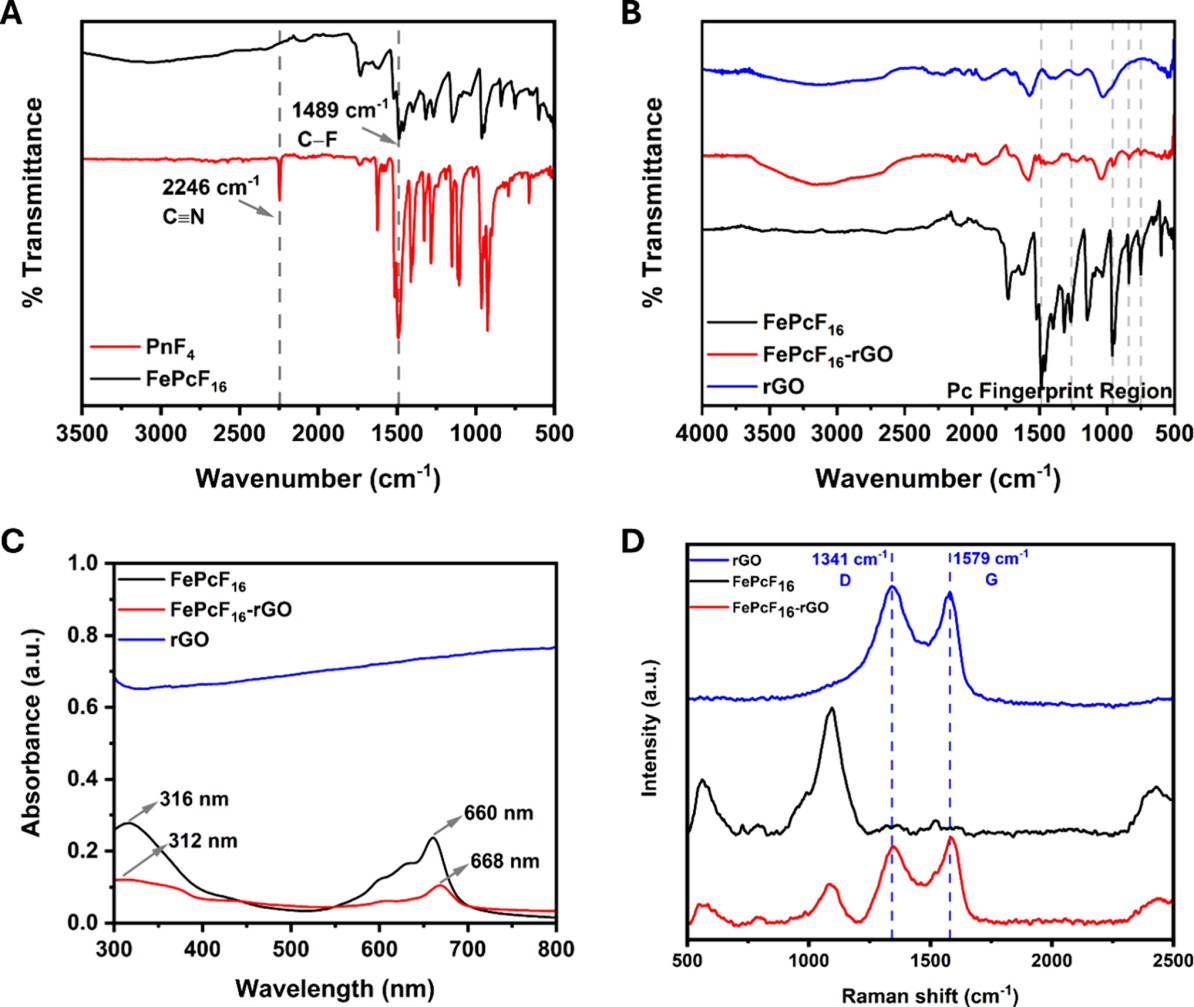
(A) FT-IR spectra of PnF_4_ and FePcF_16_. (B) FT-IR spectra of FePcF_16_, rGO, and the FePcF_16_-rGO hybrid. (C) UV–vis spectra of FePcF_16_, rGO, and the FePcF_16_-rGO hybrid in DMSO. (D) Raman spectra of FePcF_16_, rGO, and the FePcF_16_-rGO hybrid.

Figure 1A shows the FT-IR characterization that confirms the FePcF_16_ and FePcF_16_-rGO hybrid preparation. The absence of the C≡N vibration peak at 2246 cm^–1^ and the retention of the C–F at 1489 cm^–1^ indicate the formation of FePcF_16_. rGO formation is evidenced by observing characteristic vibrational peaks at 3201 cm^–1^ (O–H) and 1581 cm^–1^ (C=C) in the FT-IR spectrum. These peaks are maintained during the synthesis of the FePcF_16_-rGO hybrid, as seen in Figure 1B.

Absorption spectroscopy is a powerful tool for identifying different MPc complexes and understanding their electronic arrangement. Employing absorption spectroscopy enables us to determine the effect of switching the metal center or having different substituents on the different positions of the MPc. MPc complexes typically exhibit two characteristic bands in their absorption spectra: the Q-band (600 to 750 nm) and the B-band (300 to 450 nm). These bands provide valuable information about the electronic transitions within the Pc macrocycle; the Q-band generates from π → π* transitions from orbitals a_1u_ to e_g_*, and the B-band generates from a_2u_ to e_g_*orbitals.^44^Figures S1 and S2 show the spectrum of the PnF_4_ and the unsubstituted FePc compared with the FePcF_16_, respectively. Figure S2 compares the unsubstituted FePc and FePcF_16_, showing characteristic π → π* transitions for the B-band at 322 and 316 nm, respectively, and Q-band transitions in the visible region at 654 and 660 nm, respectively. The Q-band of the FePcF_16_ is red-shifted compared to the unsubstituted FePc, due to the substituents reducing the HOMO–LUMO energy gap of the phthalocyanine ring.^45^Figure 1C shows the UV–vis spectrum of the rGO, FePcF_16_, and FePcF_16_-rGO hybrid in DMSO (10^–5^ M). The lack of absorption peaks in the UV–vis spectrum of rGO in DMSO solution is likely due to poor dispersion of the rGO. FePcF_16_ showed characteristic π → π* transitions at 316 nm (B-band) and 660 nm (Q-band) in their absorption spectra. The FePcF_16_-rGO hybrid exhibited similar transitions, with the B-band at 312 nm and the Q-band slightly red-shifted to 668 nm. The FePcF_16_-rGO hybrid exhibits a red-shifted and broadened Q-band absorption (8 nm) compared to FePcF_16_, signifying a charge transfer from FePcF_16_ to rGO and a reduced HOMO–LUMO energy gap in the phthalocyanine ring due to π – π interactions between Pc and rGO.^39^

Figure 1D showcases the Raman spectra for FePcF_16_, rGO, and the FePcF_16_-rGO hybrid. In the rGO spectrum, the signature G band (1579 cm^–1^) arises from the in-plane vibration of sp^2^ bonded carbon atoms, indicative of the ordered graphitic structure.^46^ On the other hand, the D band (1341 cm^–1^) originates from defects and disorder within the sp^2^ network, such as vacancies, edges, or sp^3^ hybridized carbon atoms.^47,48^ In the hybrid spectrum, we observe a shift in both the G and D peaks compared to those in pure rGO, with the G band shifting by 7 cm^–1^ and the D band by 8 cm^–1^. These shifts strongly suggest an electron transfer interaction between the FePcF_16_ molecules and the rGO sheets, potentially influencing the electronic properties and enhancing the sensitivity of the hybrid material for gas sensing applications.^49^

**Figure 2:**
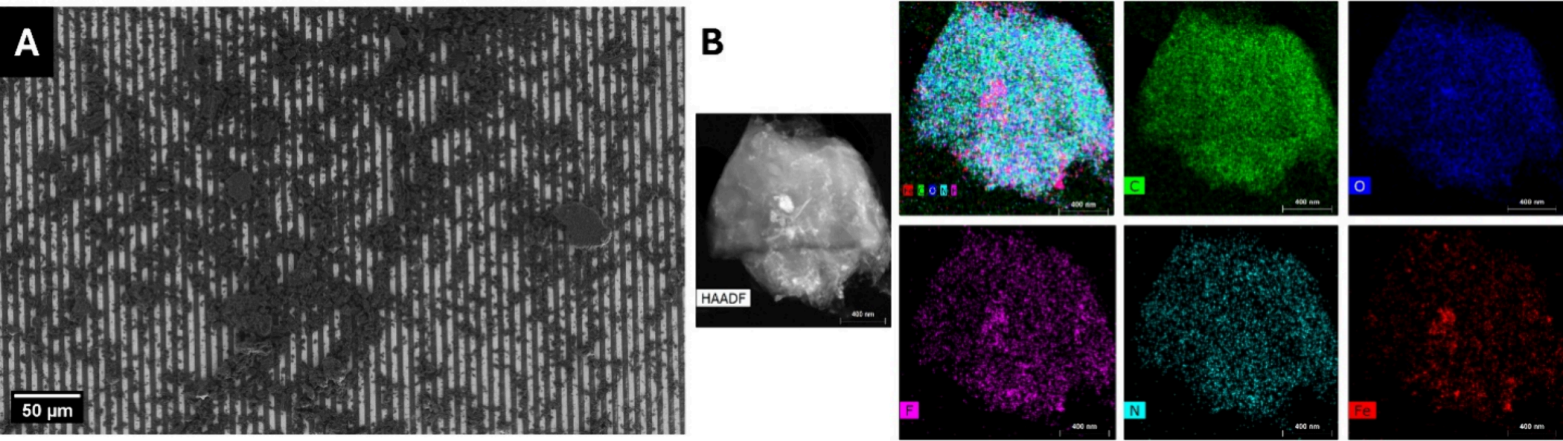
(A) SEM images of the IDE sensor based on the FePcF_16_-rGO hybrid. (B) High-angle annular dark field (HAADF) scanning transmission electron microscopy (HAADF-STEM) image and EDS elemental mappings of the FePcF_16_-rGO hybrid.

SEM analysis was employed to investigate the morphology and distribution of various materials across different electrode configurations. Figure 2A and Figure S3 showcase the uniform distribution of the FePcF_16_-rGO hybrid across an interdigitated electrode and powder form, respectively. This well-dispersed network facilitates efficient electron transport and gas diffusion and maximizes the exposure of active sites, contributing to improved sensing performance. Moreover, TEM analysis of the FePcF_16_-rGO hybrid shows a highly interconnected structure where the FePcF_16_ are homogeneously dispersed on the rGO sheets. High-resolution TEM images in Figure S4 confirm the nanoscale interaction between FePcF_16_ and rGO, hence the intimate contact for efficient charge transfer.

Energy-dispersive X-ray spectroscopy (EDS) analysis confirmed the presence of FePcF_16_ in the rGO hybrid powder (Figure S5 and Table S1). The elemental composition of FePcF_16_-rGO hybrid was determined to be mass% values of N (21.16085%), O (36.73014%), Fe (24.68725%), and F (17.42176%). The presence of Fe, N, and F confirms the successful synthesis of the hybrid material. The carbon percentage was not included in the analysis because the base of the TEM support grid consists of a copper and carbon base that contributes a lot to the detected carbon signal. Therefore, it is not easy to give the exact amount of carbon coming from the sample due to the overwhelming contribution of the grid. Elemental mapping, as shown in Figure 2B, further corroborates the distribution of these elements within the hybrid material.

### Gas Sensing Properties of the FePcF_16_-rGO Hybrid

The gold IDEs (ED-IDE3-Au) are obtained from MicruX Technologies with the following features: the gap between digits is 5 μm, and the number of digits is 180 pairs with a width of 5 μm. FePcF_16_-rGO is deposited onto IDEs using a drop-coating method to fabricate a chemiresitive gas sensor. The FePcF_16_-rGO hybrid is dispersed in ethanol by sonication treatment. The dispersed material is then drop-cast onto the IDE surface using the drop-casting technique and dried to form the film that connects the IDE pairs. The SEM image (Figure 2A) shows a close-up view of FePcF_16_-rGO materials deposited on the substrate, forming continuous pathways bridging the IDE. The gas sensing experiments were conducted within a controlled environment using the gas sensing system illustrated in Figure S6. Before gas testing, the device’s functionality was verified by obtaining its I–V curves under a bias voltage. The device is connected, and compressed N_2_ gas is injected into the chamber and purged for 10 min to ensure consistent signal output from the devices. The results are shown in Figure S7 by applying voltages between +7 V and −7 V, exhibiting a nonlinear (semiconductor) behavior of the I–V curve of FePcF_16_-rGO hybrid on the IDE.

The gas sensing properties of the FePcF_16_-rGO hybrid were evaluated using NO_2_ as the target gas and diluted with N_2_. The characteristic dynamic response curves of sensing devices utilizing FePcF_16_ and rGO when exposed electrical conductivity compared to its FePcF_16_ counterpart, aligning with the I–V curve test findings. Both the FePcF_16_-based sensor and the rGO sensor exhibited responsiveness to NO_2_. However, it is essential to note that neither material could recover after the initial detection cycle, indicating they are susceptible to poisoning, which aligns with the reported literature.^50^ In contrast, the FePcF_16_-rGO hybrid gas sensor, as illustrated in Figure S5, demonstrates superior performance with enhanced response and recovery compared to its individual components.

**Figure 3:**
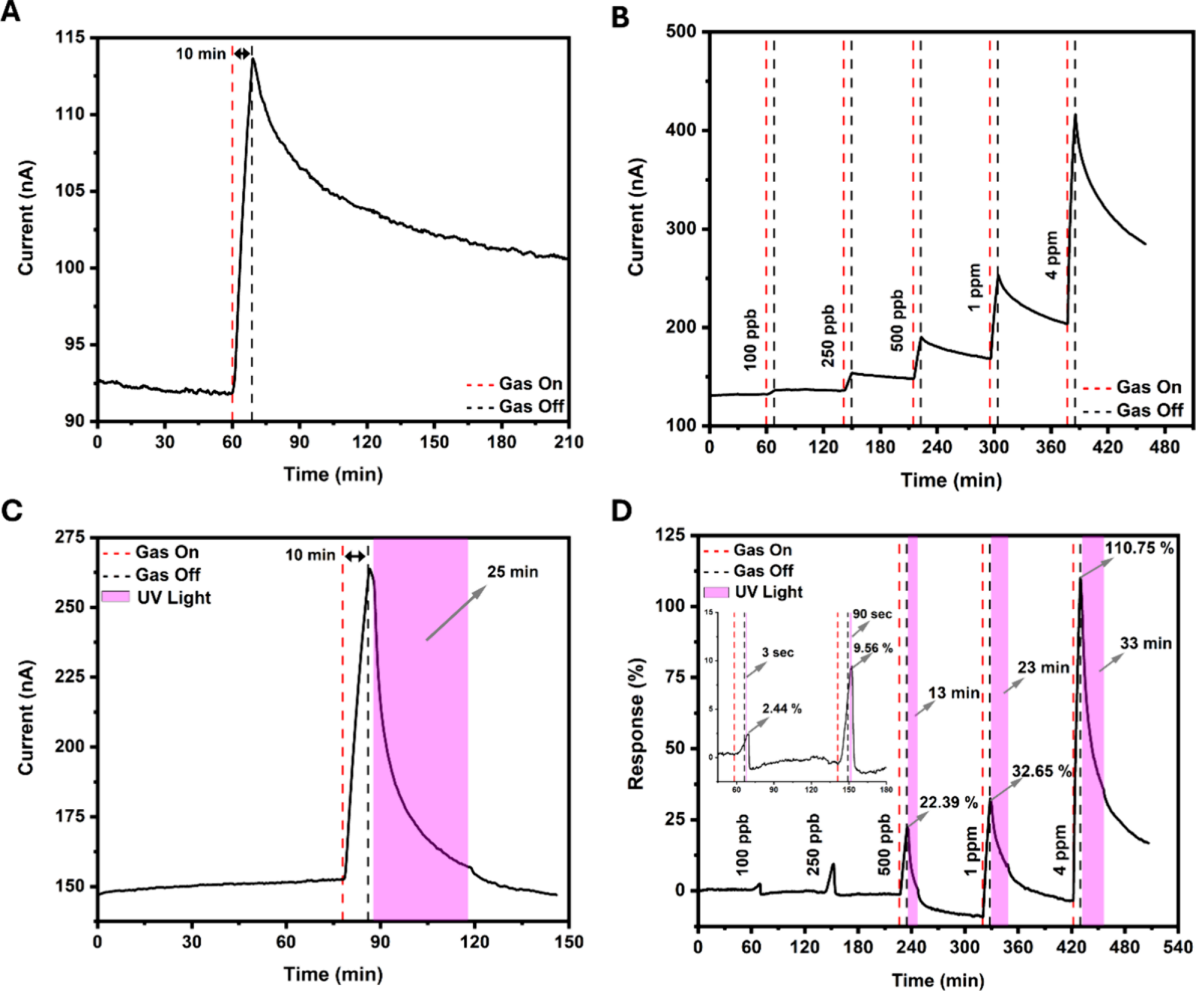
(A) Dynamic current characteristic curve of the FePcF_16_-rGO hybrid sensor exposed to 500 ppb NO_2_ (without UV irradiation). (B) Dynamic current characteristic curve of the FePcF_16_-rGO hybrid sensor exposed to different concentrations of NO_2_ (0.1, 0.25, 0.5, 1.0, and 4 ppm) without UV irradiation. (C) Dynamic current characteristic curve of the FePcF_16_-rGO hybrid sensor exposed to 1 ppm NO_2_ (recovered with UV irradiation). (D) Dynamic response characteristic curve of the FePcF_16_-rGO hybrid sensor exposed to different concentrations of NO_2_ (0.1, 0.25, 0.5, 1.0, and 4 ppm) with UV irradiation recovery.

The exposure time of the NO_2_ gas was fixed at 10 min to facilitate comparative sensing experiments. In Figure 3A, we can observe that the FePcF_16_-rGO hybrid gas sensor responds well to 500 ppb NO_2_. The device demonstrated p-type semiconductor characteristics, wherein the sensor’s current increased upon exposure to oxidizing gas NO_2_ and subsequently recovered in N_2_ despite the hybrid composition involving n-type (FePcF_16_) and p-type (rGO) materials.^51^ In Figure 3B, the sensor could sense different concentrations of NO_2_ from 100 ppb to 4 ppm. However, the sensor will not recover independently after >120 min in N_2_. UV light addressed the recovery issue observed in the gas sensors. Leveraging the photosensitivity properties of phthalocyanine, UV illumination significantly improved the recovery performance of the FePcF_16_-rGO hybrid sensor.^52,53^ The recovery time of the FePcF_16_-rGO hybrid sensor was investigated upon UV light activation. After the NO_2_ exposure, the gas flow was switched off, and the UV light was turned on 1 min after stopping the NO_2_ flow to stabilize the sensor before switching on the UV-assisted recovery process. The recovery time was the time it took the sensor signal to achieve a steady state after the UV light was turned on. As expected, illuminating the FePcF_16_-rGO hybrid increased its carrier concentration, enhanced conductivity, and achieved a gas desorption effect.^54^ As shown in Figure 3C, the FePcF_16_-rGO hybrid sensor exhibits significant recovery after exposure to NO_2_ (1 ppm), with the current increasing due to the p-type semiconductor properties mentioned previously and recovering upon UV irradiation. In Figure 3D, the FePcF_16_-rGO hybrid sensor was exposed to 0.1, 0.25, 0.5, 1, and 4 ppm NO_2_. The sensor demonstrated a remarkable response to the different concentrations of NO_2_, with values of 2.44, 9.56, 22.39, 32.65, and 110.75%, respectively. In addition, each of the concentrations recovered when exposed to UV irradiation due to the previously mentioned statement. Recovery, facilitated by UV irradiation, varied with concentration, ranging from 0.05 to 33 min, as shown in Table S2.

**Figure 4:**
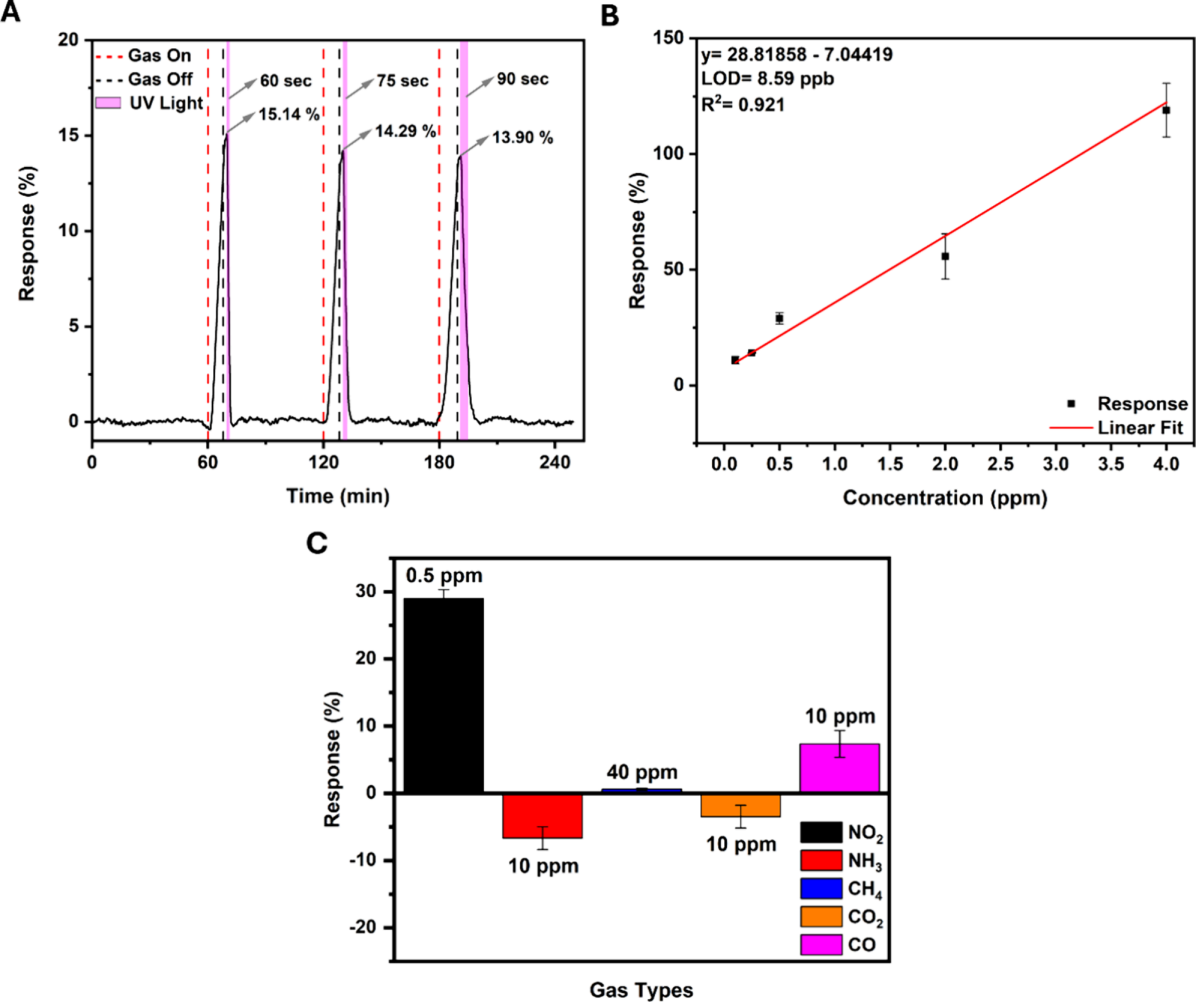
(A) Dynamic response characteristic curve of the FePcF_16_-rGO hybrid sensor exposed to three cycles of 100 ppb NO_2_. (B) Linear fit of the response of the FePcF_16_-rGO hybrid sensor to the concentration NO_2_. (C) Response of FePcF_16_-rGO hybrid-based sensor to NO_2_ and other gases.

Reproducibility is a crucial aspect of gas sensor evaluation. To assess the sensor’s reliability in detecting NO_2_, we exposed it to a known concentration of NO_2_ of 100 ppb for multiple cycles. This repetitive testing allowed us to consistently analyze the sensor’s ability to detect the gas across exposures. We monitored the sensor’s response (signal change) and recovery time between exposures to evaluate its performance. As mentioned, the same exposure and recovery procedure was repeated to evaluate the sensor’s reusability. Figure 4A shows the sensor’s response across three cycles, with 15.14, 14.29, and 13.90% values, indicating a stable and reproducible performance. This reproducible response at the ppb NO_2_ level highlights the sensor’s potential for practical applications due to its reusability.

Additionally, a linear fit of the response data is used to determine the theoretical limit of detection (LOD) for NO_2_. The LOD refers to the minimum concentration of an analyte within a sensing element that can be reliably detected with a specified probability. Each response was measured three times to obtain an average, ensuring reliability and accuracy. The determination of LOD is given by eqs 3 and 4:^55,56^
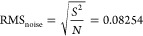
3
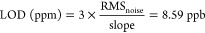
4

RMS_noise_ is the root-mean-square-standard deviation of the noise, which is calculated to be equal to 0.08254 based on 290 data points (*N*) and a standard deviation (*S*) of 1.40562 from the baseline of the curve for the FePcF_16_-rGO hybrid sensor, fitting to eq 3. Derived from the calculated RMS_noise_, the theoretical limit of detection (LOD) for the FePcF_16_-rGO hybrid sensor is determined to be 8.59 ppb (Figure 4B) at a signal-to-noise ratio of 3, as per eq 4.

Further study of our device was done by exposing the sensor to various gases to test its selectivity, which is a significant characteristic of gas sensors. The selectivity of our gas sensor was determined by exposing the device individually to other types of gases NH_3_, methane (CH_4_), carbon dioxide (CO_2_), and carbon monoxide (CO)-at room temperature, as shown in Figure 4C. The reproducibility of the results was checked by testing each gas individually three times. The FePcF_16_-rGO hybrid sensor exhibited good selectivity toward 0.5 ppm NO_2_ at 28.9%. The device did not show significant responses to NH_3_ and CO_2_ at low concentrations, proving its ability to distinguish NO_2_ from these potential interferents. In the case of NH_3_ and CO_2_, the response was negative since the device is a p-type semiconductor, and the current will decrease in the case of exposure to a reducing gas. However, the device showed a −6.7 and −3.5% response, respectively, only at higher concentrations. For CH_4_ and CO, the response was sluggish even at higher concentrations, showing a response of 0.6 and 7.3%, respectively. Compared to other reported NO_2_ sensors, especially those based on similar materials, as shown in Table S3, the FePcF_16_-rGO sensor exhibits competitive performance. Although some sensors show higher sensitivity or lower detection limits, the FePcF_16_-rGO hybrid sensor demonstrates a balanced combination of high response, a low theoretical detection limit (8.59 ppb), and recovery under UV light-assisted operation. This combination makes it a promising candidate for practical NO_2_ detection applications, particularly where fast response and recovery times are required. This indicates that the FePcF_16_-rGO sensor has higher selectivity for NO_2_ at lower concentrations, which gives a strong and distinguishable response even at low levels of NO_2_, while it only slightly responds to other gases even when their concentrations are increased.

### Gas Sensing Mechanism

The FePcF_16_-rGO hybrid sensor exhibits remarkable gas sensing capabilities due to the enhanced functionality of the combination of FePcF_16_ and rGO, facilitating complementary interactions with target gas molecules. Additionally, the FePcF_16_-rGO hybrid’s p-type behavior stems from the interplay between the p-type semiconductor characteristics of rGO and the n-type semiconductor characteristics of FePcF_16_.^35,50^ The role of rGO in changing the electronic properties of the material toward improved conductivity and enhanced interactions with NO_2_ molecules has been proven in similar materials to be the result of a combination of multiple factors, such as (i) restoration of conductive network from the removed oxygen-containing groups, regenerating the sp^2^-bonded carbon network, (ii) allowing electron mobility, and (iii) providing high surface area for contact with gas molecules, thus improving interfacial interactions.^57−59^ This unique combination of properties and interactions suggests that the gas sensing goes as follows:

The sensor’s pre-exposure to atmospheric oxygen during fabrication leads to both physical and chemical adsorption of oxygen molecules onto the surface of the hybrid (eq 5)^60,61^
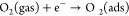
5

The preadsorbed oxygen molecules can withdraw electrons from the hybrid surface, inducing a slight p-type character in the rGO. The following reaction processes can represent this electron transfer:
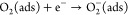
6
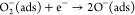
7
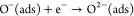
8

As the sensor encounters NO_2_ gas, the interaction between the gas molecules and the preadsorbed oxygen species on the p-type hybrid layer triggers a complex series of electron transfer reactions (shown in eqs 9 and 10).^62,63^ These reactions involve NO_2_ trapping electrons from both the hybrid material and the preadsorbed oxygen species. This electron transfer process increases the concentration of holes (majority carriers in p-type material) within the rGO, leading to a rise in the sensor’s conductivity.^64^
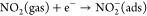
9
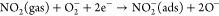
10

After the NO_2_ flow is stopped and the remaining gas is purged from the sensing environment, the hybrid sensor is irradiated with UV light. This process takes advantage of the FePcF_16_’s photosensitivity and photogenerated electron–hole pairs within the material (eq 11)^65^
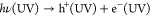
11

During UV-aided recovery, photogenerated carriers facilitate the desorption of remaining NO_2_ from the sensor surface, restoring its baseline conductivity.^66^ One potential mechanism involves recombining photogenerated holes with the adsorbed NO_2_ molecules, leading to their neutralization and subsequent desorption (eq 12).^67,68^
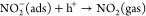
12

Furthermore, the UV photosensitivity of FePcF_16_ allows for sensor recovery, with hole recombination potentially playing a significant role in the desorption of NO_2_, ultimately restoring the baseline conductivity.

## Conclusions

In conclusion, the FePcF_16_-rGO hybrids were effectively synthesized through noncovalent interactions, where the FePcF_16_ is functionalized on the surface of the rGO. This preserves the structural integrity and surface area of rGO and provides active sites for NO_2_ adsorption by FePcF_16_. The unique large-surface-area structure facilitated NO_2_ diffusion, coupled with abundant active sites for NO_2_ adsorption and excellent conductivity for efficient electron transport. As a result, the sensors exhibited remarkable sensitivity and linear response-concentration characteristics toward NO_2_ at ambient temperature. UV light irradiation effectively addressed recovery issues in the sensors, offering a practical solution for improved performance. Notably, the FePcF_16_-rGO hybrid sensor shows remarkable sensitivity toward low concentrations of NO_2_ with a 15.14% response for 100 ppb and a quick recovery time when irradiated with UV light of 60 s. Moreover, our investigation into the sensor’s selectivity revealed its robust response to NO_2_ while maintaining minimal interference from other gases and can reach an LOD of 8.59 ppb NO_2_.

The outstanding gas sensing response of the FePcF_16_-rGO hybrid sensor originates from the complementary interactions between the composite material and target gas molecules, enabled by the synergistic effects of FePcF_16_ and rGO. Additionally, the FePcF_16_-rGO hybrid’s p-type behavior stems from the interplay between the p-type semiconductor characteristics of rGO and the n-type semiconductor characteristics of FePcF_16_. This unique combination suggests a complex gas sensing mechanism involving oxygen adsorption, electron transfer reactions with NO_2_, and UV-aided desorption.

## Supporting Information Available

Additional UV–vis spectra, SEM and HRTEM images, and HAADF-STEM image and EDS analysis of the materials used in the sensor fabrication; normalized quantitative weight percentages for the elements in FePcF_16_-rGO hybrid; schematic illustration of the gas sensing facility; characterization of the ohmic behavior of the gas sensor; dynamic response curves of the device; summary tables of the response and recovery times and sensor comparison (PDF)
