# Supplementary material for: High-Sensitivity NO2 Gas Sensor: Exploiting UV-Enhanced Recovery in a Hexadecafluorinated Iron Phthalocyanine-Reduced Graphene Oxide
Source: ACS Omega. 2025 Jan 16;10(3):2809–18. doi: 10.1021/acsomega.4c08662 (PMC11780449; doi:10.1021/acsomega.4c08662)
Supplement: Supplementary file 1 — ao4c08662_si_003.pdf [file ao4c08662_si_003.pdf]

Supporting Information

High-Sensitivity NO_2 Gas Sensor: Exploiting UV-Enhanced Recovery in a Hexadecafluorinated Iron Phthalocyanine-Reduced Graphene Oxide

John A. Cruz Lozada[†,‡], Ricardo A. Rosario[†], Soraya Y. Flores[†], Kim Kisslinger, Luis F. Fonseca[†] and Dalice M. Piñero Cruz[†,‡,*]

[†]Faculty of Natural Sciences, University of Puerto Rico, Río Piedras Campus, San Juan 00931, Puerto Rico; john.cruz1@upr.edu; soraya.flores@upr.edu; luis.fonseca@upr.edu

[‡]Molecular Science Research Center, San Juan 00926-2614, Puerto Rico

[#]Brookhaven National Laboratory, Center for Functional Nanomaterials, Bldg 735, Upton, NY 11973-5000

[*]e-mail address of the corresponding author: dalice.pinero@upr.edu

Table of Contents:

Figure S1. UV-vis spectra of $FePcF_{16}$ and PnF_4 in DMSO.

Figure S2. UV-vis spectra of $FePcF_{16}$ and FePc in DMSO.

Figure S3. Scanning electron microscope (SEM) images of $FePcF_{16}$-rGO hybrid (A) powder and (B) deposited on the interdigitated electrode (IDE).

Figure S4. HRTEM image of the $FePcF_{16}$-rGO hybrid material. Lattice fringes could be seen, confirming the crystalline nature of both rGO sheets and $FePcF_{16}$. The homogenous dispersion of $FePcF_{16}$ on the rGO surface underlines the interaction between both components, necessary for the effective charge transfer and maximum exposure of the active sites.

Figure S5. HAADF-STEM image and EDS spectrum for the $FePcF_{16}$-rGO hybrid material. Elemental mappings of Fe (red), C (green), O (blue), N (yellow), and F (magenta) confirm the homogeneous distribution of these elements in the material. Copper (Cu) peaks were omitted from the analysis as they originate from the TEM grid. The table summarizes the normalized weight and atomic concentrations of the detected elements.

Table S1. Normalized quantitative weight percentages for the elements in $FePcF_{16}$-rGO hybrid, with carbon omitted due to interference from the carbon support film

Figure S6. Schematic illustration of the gas sensing facility.

Figure S7. I-V response graph of the $FePcF_{16}$-rGO hybrid sensor in a positive and negative voltage.

Figure S7. Dynamic response characteristic curve of the $FePcF_{16}$, rGO, and $FePcF_{16}$-rGO hybrid sensors exposed to 500 ppb NO_2.

Table S2. Response and recovery time of $FePcF_{16}$-rGO hybrid sensor for NO_2

Table S3. Comparative analysis of $FePcF_{16}$-rGO hybrid and recent rGO-based hybrids for sensing applications

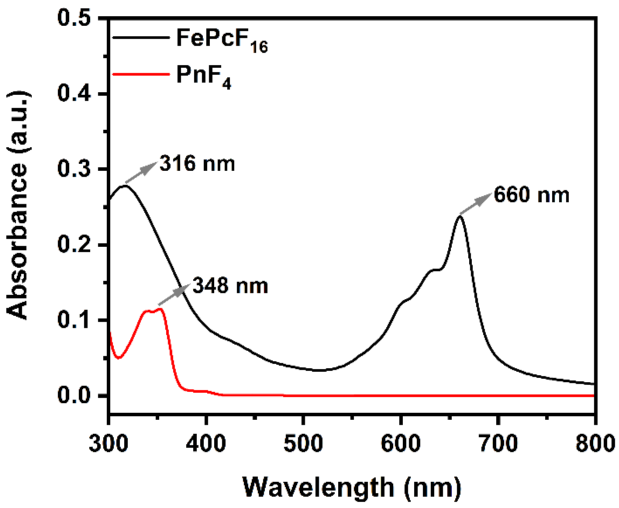

Figure S1. UV-vis spectra of $FePcF_{16}$ and PnF_4 in DMSO.

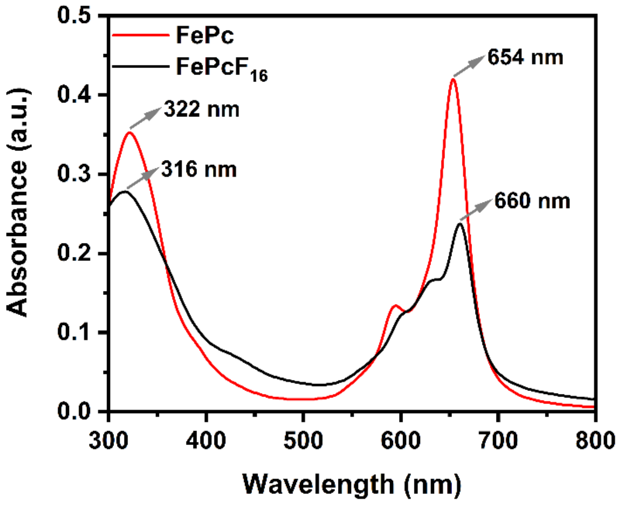

Figure S2. UV-vis spectra of $FePcF_{16}$ and FePc in DMSO.

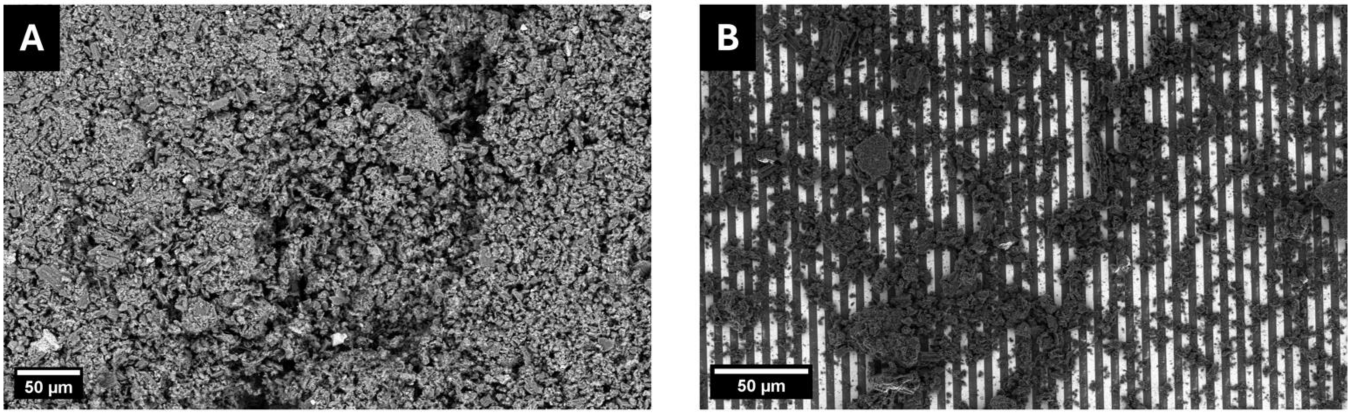

Figure S3. Scanning electron microscope (SEM) images of $FePcF_{16}$-rGO hybrid (A) powder and (B) deposited on the interdigitated electrode (IDE).

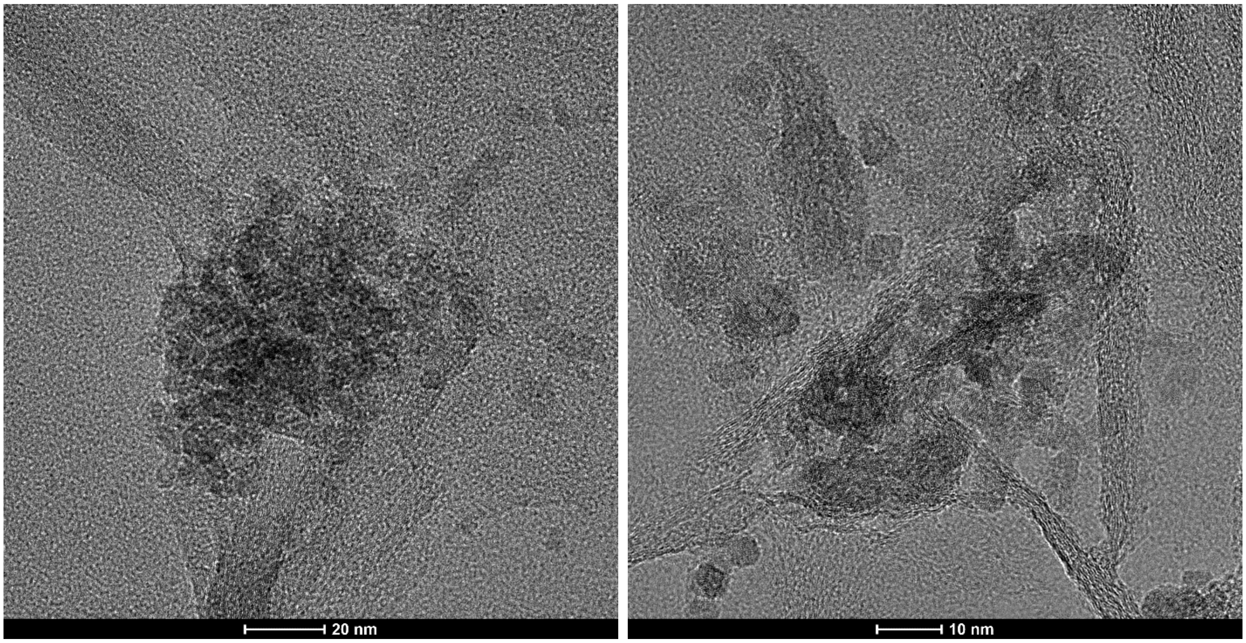

Figure S4. HRTEM image of the $FePcF_{16}$-rGO hybrid material. Lattice fringes could be seen, confirming the crystalline nature of both rGO sheets and $FePcF_{16}$. The homogenous dispersion of $FePcF_{16}$ on the rGO surface underlines the interaction between both components, necessary for the effective charge transfer and maximum exposure of the active sites.

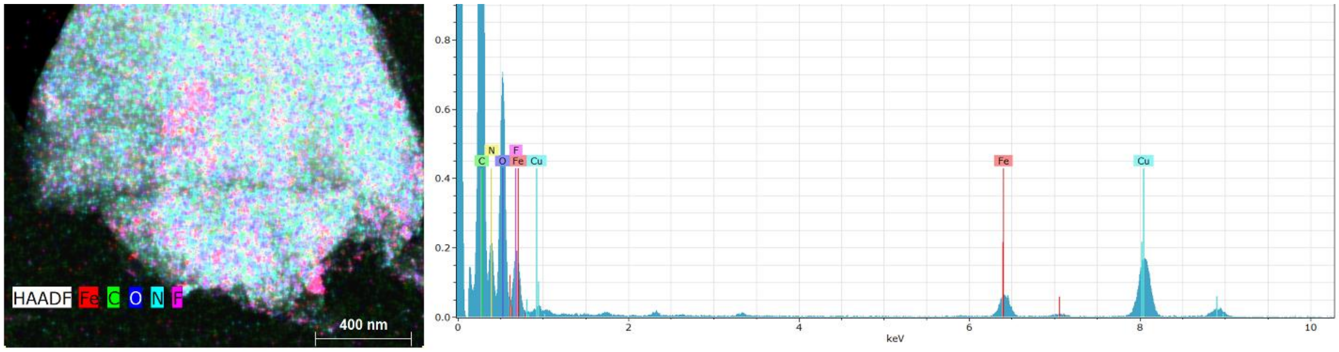

Figure S5. HAADF-STEM image and EDS spectrum for the $FePcF_{16}$-rGO hybrid material. Elemental mappings of Fe (red), C (green), O (blue), N (yellow), and F (magenta) confirm the homogeneous distribution of these elements in the material. Copper (Cu) peaks were omitted from the analysis as they originate from the TEM grid. The table summarizes the normalized weight and atomic concentrations of the detected elements.

Table S1. Normalized quantitative weight percentages for the elements in $FePcF_{16}$-rGO hybrid, with carbon omitted due to interference from the carbon support film

Element	Series	Net	[wt.%]	[norm. wt.%]	[norm. at.%]	Error in wt.% (3 Sigma)
**Iron**	**K-series**	**4646**	**24.68725**	**24.68725**	**8.557687**	**2.582254**
**Nitrogen**	**K-series**	**4484**	**21.16085**	**21.16085**	**29.24696**	**2.20239**
**Oxygen**	**K-series**	**9562**	**36.73014**	**36.73014**	**44.4429**	**3.567499**
**Fluorine**	**K-series**	**4593**	**17.42176**	**17.42176**	**17.75246**	**1.822352**
		**Sum:**	**100**	**100**	**100**	

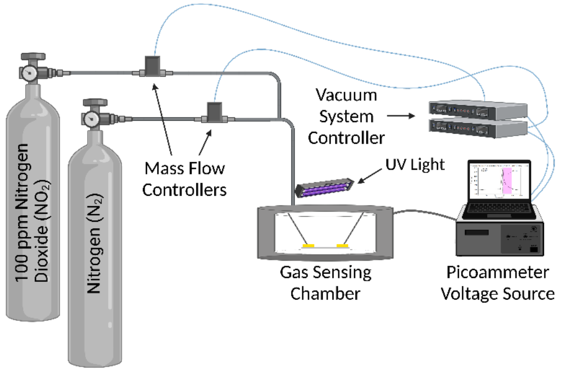

Figure S6. Schematic illustration of the gas sensing facility.

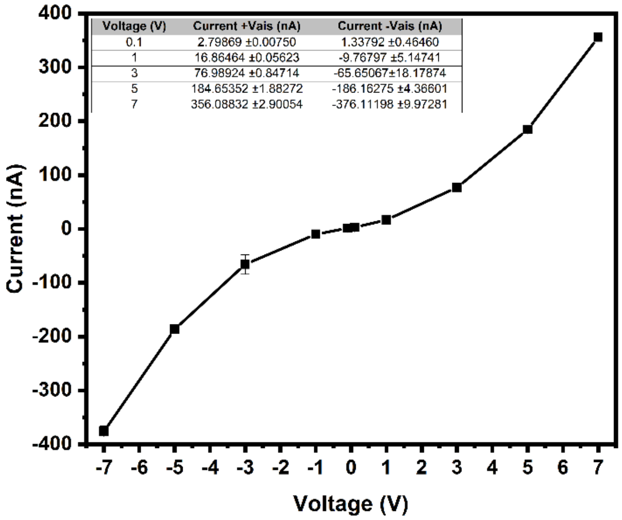

Voltage (V)	Current +Vais (nA)	Current -Vais (nA)
0.1	2.79869 ±0.00750	1.33792 ±0.46460
1	16.86464 ±0.05623	-9.76797 ±5.14741
3	76.98924 ±0.84714	-65.65067±18.17874
5	184.65352 ±1.88272	-186.16275 ±4.36601
7	356.08832 ±2.90054	-376.11198 ±9.97281

Figure S7. I-V response graph of the $FePcF_{16}$-rGO hybrid sensor in a positive and negative voltage.

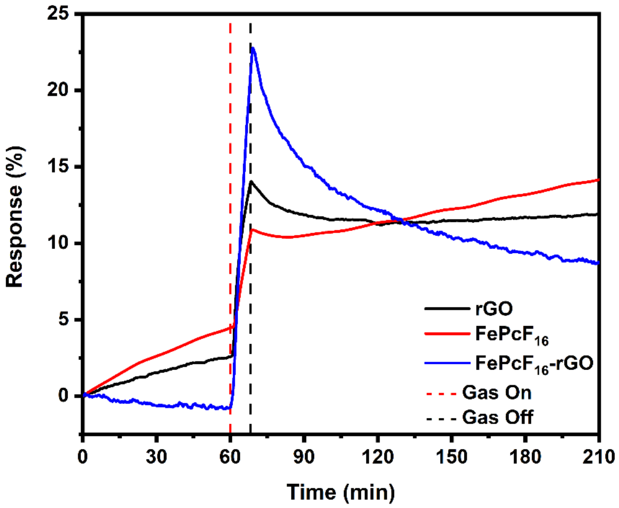

Figure S8. Dynamic response characteristic curve of the $FePcF_{16}$, rGO, and $FePcF_{16}$-rGO hybrid sensors exposed to 500 ppb NO_2.

Table S2. Response and recovery time of $FePcF_{16}$-rGO hybrid sensor for NO_2

Concentration (ppm)	Response (%)	Recovery (%)	Recovery Time (min)
0.1	2.44	82.03	0.05
0.25	9.56	80.29	1.5
0.5	22.39	96.75	13
1	32.65	70.28	23
4	110.75	70.42	33

Table S3. Comparative analysis of $FePcF_{16}$-rGO hybrid and recent rGO-based hybrids for sensing applications

Material	Sensor Preparation	Concentration Range (ppm)	Response	Detection Limit (ppm)	References
PBTTT/GO	Spreading-Solidifying Method	1-30	174% (10 ppm)	0.37	1
V_2CTx/SnS_2	Drop-Cast	0.4-5	149.22% (5 ppm)	0.30	2
rGO/$Ti_3C_2T_x$	Drop-Cast	10-100	37% (50 ppm)	10	3
P_3HT/rGO	Shear coating-assisted phase separation	0.5-50	61.3% (10ppm)	0.5	4
WO_3/S-rGO	Drop-Cast	1-50	149.5% (20 ppm)	0.25	5
rGO/Fe_2O_3	Layer-by-Layer Coating	1-20	≈26% (4 ppm)	Not mention	6
SnO_2/rGO	Drop-Cast	1-80	2.99% (80 ppm)	0.209	7
$FePcF_{16}$-rGO	Drop-Cast	0.100-4	110.75% (4 ppm)	0.00859	This Report

References

(1) Sahu, P. K.; Pandey, R. K.; Dwivedi, R.; Mishra, V. N.; Prakash, R. Polymer/Graphene Oxide Nanocomposite Thin Film for NO2 Sensor: An in Situ Investigation of Electronic, Morphological, Structural, and Spectroscopic Properties. *Sci Rep* **2020**, *10* (1), 2981.

(2) Zhang, Y.; Li, Y.; Jiang, Y.; Duan, Z.; Yuan, Z.; Liu, B.; Huang, Q.; Zhao, Q.; Yang, Y.; Tai, H. Synergistic Effect of Charge Transfer and Interlayer Swelling in V2CTx/SnS2 Driving Ultrafast and Highly Sensitive NO2 Detection at Room Temperature. *Sensors and Actuators B: Chemical* **2024**, *411*, 135788.

(3) Tran, N. M.; Ta, Q. T. H.; Noh, J.-S. rGO/Ti3C2Tx Heterostructures for the Efficient, Room-Temperature Detection of Multiple Toxic Gases. *Materials Chemistry and Physics* **2021**, *273*, 125087.

(4) Shin, S. Y.; Jeong, G.; Phu, N. A. M. M.; Cheon, H.; Tran, V. V.; Yoon, H.; Chang, M. Improved NO2 Gas-Sensing Performance of an Organic Field-Effect Transistor Based on Reduced Graphene Oxide-Incorporated Nanoporous Conjugated Polymer Thin Films. *Chem. Mater.* **2023**, *35* (18), 7460–7474.

(5) Wang, T.; Hao, J.; Zheng, S.; Sun, Q.; Zhang, D.; Wang, Y. Highly Sensitive and Rapidly Responding Room-Temperature NO2 Gas Sensors Based on WO3 Nanorods/Sulfonated Graphene Nanocomposites. *Nano Res.* **2018**, *11* (2), 791–803.

(6) Pisarkiewicz, T.; Maziarz, W.; Małolepszy, A.; Stobiński, L.; Michoń, D. A.; Szkudlarek, A.; Pisarek, M.; Kanak, J.; Rydosz, A. Nitrogen Dioxide Sensing Using Multilayer Structure of Reduced Graphene Oxide and α-Fe2O3. *Sensors* **2021**, *21* (3), 1011.

(7) Verma, M.; Bahuguna, G.; Shukla, S.; Gupta, R. SnO2 Nanoparticle-Reduced Graphene Oxide Hybrids for Highly Selective and Sensitive NO2 Sensors Fabricated Using a Component Combinatorial Approach. *ACS Appl. Nano Mater.* **2022**, *5* (12), 19053–19061.
